# Seagrass ecosystems reduce disease risk and economic loss in marine farming production

**DOI:** 10.1073/pnas.2416012121

**Published:** 2024-12-16

**Authors:** Evan A. Fiorenza, Nur Abu, William E. Feeney, Steven R. Limbong, Claire B. Freimark, Jamaluddin Jompa, C. Drew Harvell, Joleah B. Lamb

**Affiliations:** ^a^Department of Ecology and Evolutionary Biology, University of California, Irvine, CA 92697; ^b^Department of Environmental Engineering, Muhammadiyah University of Sorong, Sorong 98416, Indonesia; ^c^Doñana Biological Station, Spanish National Research Council, Seville 41092, Spain; ^d^Ministry of National Development Planning, Government of Indonesia, Jakarta 10310, Indonesia; ^e^Faculty of Marine and Fisheries Sciences, Hasanuddin University, Makassar 90245, Indonesia; ^f^Department of Ecology and Evolutionary Biology, Cornell University, Ithaca, NY 14853

**Keywords:** blue economy, natural capital, sustainable development, coastal ecosystems, marine resources

## Abstract

Seaweed farming comprises over half of global coastal and marine aquaculture production by mass; however, the future of the industry is increasingly threatened by disease outbreaks. Nature-based solutions provided by enhancing functions of coinciding species or ecosystems offer an opportunity to increase yields by reducing disease outbreaks while conserving biodiversity. Seagrass ecosystems can reduce the abundance of marine bacterial pathogens, although it remains unknown whether this service can extend to reducing disease risk in a marine resource. Using a meta-analysis of articles published over the past 40 y, we find that 17 known diseases of seaweeds are attributed to bacteria that have been previously shown to be lower when associated with seagrass ecosystems. Next, we surveyed over 8,000 individual seaweeds among farms in Indonesia and found that disease risk is reduced by 75% when seaweeds are co-cultivated directly within seagrass ecosystems, compared to when seagrass ecosystems were removed. Finally, we estimate that farming seaweed with seagrass ecosystems could increase annual revenue by $292,470 – $1,015,990 USD per km^2^ from yield loss due to disease reduction and that ~20.7 million km^2^ in 107 countries and 34 territories have suitable environmental conditions for farming seaweeds with seagrass ecosystems. These results highlight the global utility for nature-based solutions as an ecologically and economically sustainable management strategy.

More than 35 million tons of seaweeds are harvested each year with a value of over USD $13 billion, comprising half of global marine and coastal aquaculture production by mass (1). In addition to reducing global pressures on land-use and food security (2), seaweeds represent a major source of value with vast potential for further growth through the development of products, including plastic alternatives, biofuels, and negative emission technologies (3, 4). Despite seaweed cultivation generating substantial social and economic benefits, yields are threatened by disease outbreaks (5). As seaweeds are primarily cultivated in open marine and coastal systems, traditional methods of disease management are often not effective or cost-efficient (6). Nature-based solutions—such as harnessing functions provided by coinciding species or ecosystems—may instead offer an ecologically sustainable and economically viable option. Successful examples of these facilitative interactions include applications ranging from marine food systems (7) to restoration (8). Coastal ecosystems provide filtration services that are widely used to improve water quality and health outcomes (9), with mounting evidence indicating that seagrass ecosystems reduce the relative abundance of marine bacterial pathogens and disease (7, 10, 11). Despite the globally expansive range of seagrass ecosystems (12), it remains unknown whether this ecosystem service can extend to reducing disease risk of marine and coastal aquaculture resources.

## Results

Using a meta-analytical approach, we identified 567 unique seaweed host–pathogen combinations among 215 articles over a 41-y period from 1979 to 2020. We first filtered the dataset for marine bacterial pathogens that have been associated with a reduction in relative abundance where seagrass ecosystems are present in both temperate regions (7) and tropical regions (10), which specifically include the genera *Corynebacterium, Flavobacterium, Rickettsia, Shewanella,* and *Vibrio*. From these, we identified 17 described diseases affecting 12 species of aquacultured seaweeds and 4 species of wild seaweeds (Dataset S1). In particular, 39% of countries that currently report commercially harvesting one of the most globally important farmed seaweeds by mass (*Kappaphycus*) (1) have been affected by an outbreak of a condition colloquially referred to as ice-ice disease (IID) (Dataset S2), where the suspected origin of infection is associated with opportunistic bacteria often in the genus *Vibrio*, but also in the genera *Aeromonas, Alteromonas, Bacillus, Cytophaga, Flavobacterium,* and *Pseudoalteromonas* (13).

**Fig. 1.**
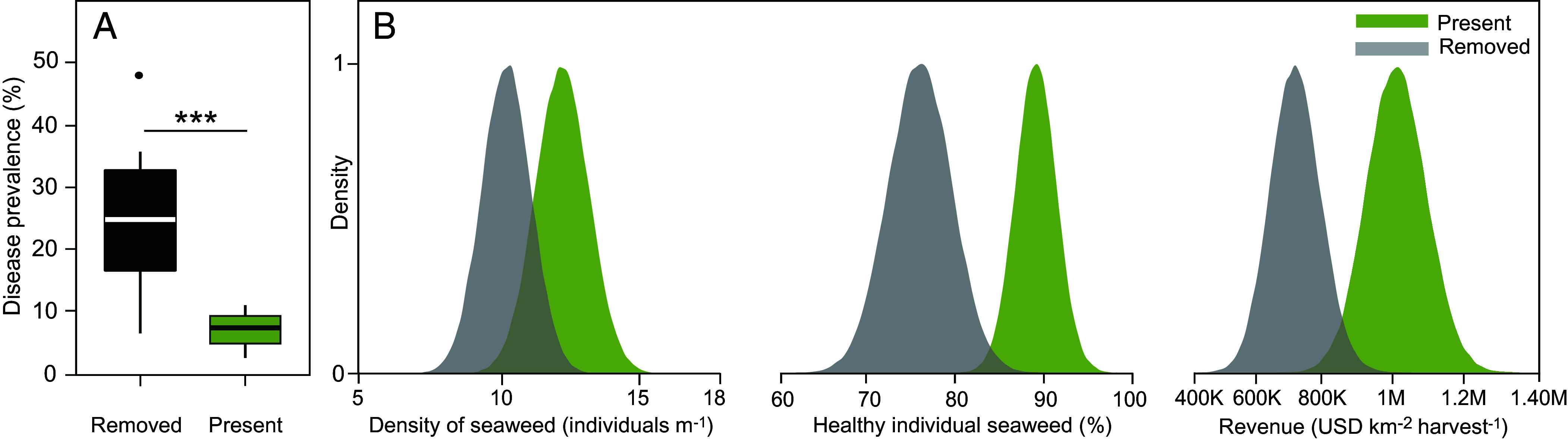
Seagrass ecosystems reduce disease risk and associated yield loss of farmed seaweeds. (*A*) Box plots (median and 50% quantile) and whisker plots (95% quantile) of disease prevalence for the seaweed *Kappaphycus* from surveys conducted among farms cultivating in locations where seagrass ecosystems were present and locations where seagrass ecosystems naturally occur, but have been removed (*n* = 8,876 individual seaweeds, 8 farms each treatment), and (*B*) density distribution plots of production potential estimated from surveys conducted among farms followed by 100,000 simulations using a normal distribution. Revenue generated per km^2^ during each harvest was determined using the simulated density (m^−1^) and simulated prevalence of healthy individuals (%) and then estimated using the mean farm gate value (market value in USD minus selling costs) of dry *Kappaphycus* available from six countries in 2015, excluding inflation and the potential for other uses.

The largest share of suitable ocean area for seaweed farming is located in the Indonesian exclusive economic zone (2) and currently the second largest global producer of farmed seaweed (1). Seagrass ecosystems are often removed for seaweed farming, providing a natural experiment to assess whether seagrass ecosystems influence disease levels in a co-cultivated marine resource. Here, we visually examined 8,876 individual seaweeds across 16 farms in Indonesia and found a 75% decrease in IID prevalence when farmers co-cultivated the seaweed *Kappaphycus* directly within seagrass ecosystems (mean ± SE = 6.3 ± 1.2%, data range = 1.2% – 14.5%) compared to where seagrass ecosystems were removed (25.1 ± 6.9%, data range = 1.0% – 79.2%, generalized linear mixed model, Z = −4.608, *P* < 0.001, Fig. 1*A*).

Progressive disintegration of tissue associated with IID can reduce seaweed yield (12). Therefore, we estimated the difference in annual revenue of farming seaweed with seagrass ecosystems from reducing yield loss. By retaining seagrass ecosystems within seaweed cultivation areas, revenue generated per harvest increases by $292,470 USD per km^2^ each year, where we estimate that seaweed farmed with seagrass ecosystems is valued at $1,015,990 ± $86,010 USD per km^2^ per harvest compared to $723,520 ± $72,840 USD per km^2^ per harvest for seaweed farmed without seagrass ecosystems (Fig. 1*B*). The number of harvests per year can vary depending on nutrient input, light, and temperature with known locations in India able to have four annual harvests and 45-d cultivation periods. Therefore, the annual increase in revenue could reach $1,169,880 USD per km^2^ with four harvests.

**Fig. 2.**
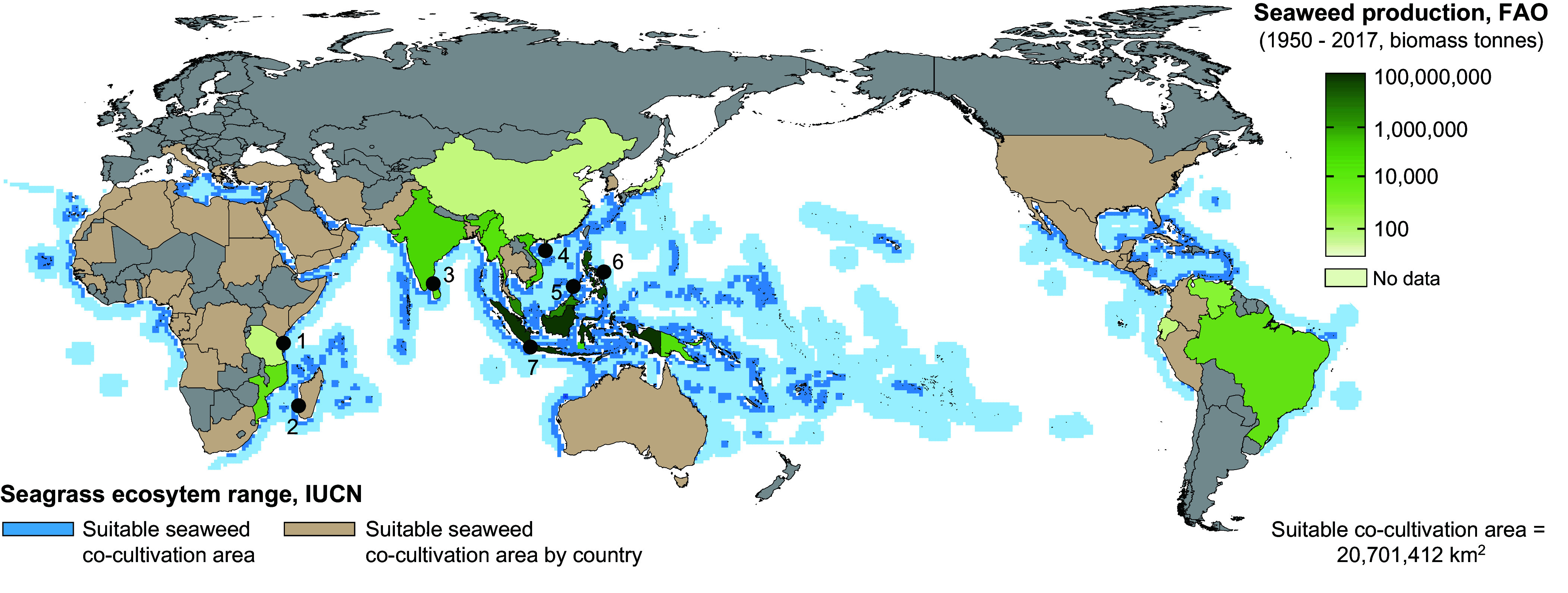
Global overlap in suitability for co-cultivating seaweed with seagrass ecosystems. Suitability range of aquaculture for the seaweed *Kappaphycus* was determined in exclusive economic zones for each country using surface nitrogen (N) and phosphorus (P) concentrations (N:P ratio < 80:1) and then restricted by the thermal tolerance limit of *Kappaphycus* using a sea surface temperature tolerance range of 20 °C to 35 °C modified from (4). Seagrass ecosystem distributions were overlaid with the suitability of seaweed aquaculture to produce an overall global suitability map. The seaweed production dataset was synthesized using global capture production of *Kappaphycus* from 1950 to 2017. Published reports of disease affecting *Kappaphycus* are indicated by numbered points and represent nine reports in seven countries.

Our global model estimates that suitable environmental conditions for cultivating *Kappaphycus* encompass 347,821,713 km^2^. From this, we find that 20,701,412 km^2^ (6.0%) overlaps with a naturally occurring seagrass ecosystem range, resulting in a potential co-cultivation area for 107 countries and 34 territories (Fig. 2). We note that the estimated co-cultivation range does not exclude areas that are protected or gazetted for other uses.

## Discussion

Farmed seaweed represents a promising approach to reduce pressures on land-use and offset atmospheric carbon dioxide emissions (2); however, scaling coastal production can come at the expense of already threatened ecosystems. Seagrass meadows are among the most threatened ecosystems globally, with rates of loss estimated as high as 7% per annum through impacts primarily associated with coastal development (14). Valuing ecosystem services can support innovative approaches for addressing challenges associated with sustainable development, although determining the full range of benefits is often limiting to these efforts. Managing disease outbreaks in intensively farmed seaweed can account for up to 50% of operating costs, with economic losses reaching US $310 million for a single outbreak (5). Here, we present evidence for a service provided by a coastal ecosystem to increase revenue by reducing disease levels in a globally important marine resource. While we are only beginning to recognize the high-value benefits provided by seagrass ecosystems and seaweed farming, coincidental services include carbon sequestration, nutrient cycling, acidification mitigation, protection from coastal erosion, and biodiversity enhancement (12, 15). Our results provide one of the largest examples of a positive facilitative interaction in a marine farming production system (8), highlighting a global opportunity for multifunctional development strategies that support a blue economy, where ecological, social, and economic benefits support conservation initiatives and sustainable development targets.

## Materials and Methods

Fieldwork was conducted at 16 seaweed farms along the southwest coast of Sulawesi in Indonesia, where seaweed is currently co-cultivated in coastal areas directly within intact seagrass ecosystems and where seagrass ecosystems naturally occur, but have been removed. The estimated economic value of disease risk reduction associated with the co-cultivation of seaweed with seagrass ecosystems and global geographic suitability is briefly described in each figure legend. Full *Materials and Methods*, including mechanisms of seagrass filtration, can be found in *SI Appendix*.

## Supplementary Material

Appendix 01 (PDF)

Dataset S01 (CSV)

Dataset S02 (CSV)

Dataset S03 (CSV)

Dataset S04 (CSV)

Dataset S05 (CSV)

Dataset S06 (CSV)

## Data Availability

R script and data files have been deposited in the Dryad Digital Repository (16).

## References

[1] FAO, The state of world fisheries and aquaculture 2022: Towards blue transformation (Food and Agriculture Organization, 2022), p. 266.

[2] S. Spillias , Reducing global land-use pressures with seaweed farming. Nat. Sustain. **6**, 380–390 (2023).

[3] Y. K. Leong, K. W. Chew, W.-H. Chen, J.-S. Chang, P. L. Show, Reuniting the biogeochemistry of algae for a low-carbon circular bioeconomy. Trends Plant Sci. **26**, 729–740 (2021). 33461869 10.1016/j.tplants.2020.12.010

[4] H. E. Froehlich, J. C. Afflerbach, M. Frazier, B. S. Halpern, Blue growth potential to mitigate climate change through seaweed offsetting. Curr. Biol. **29**, 3087–3093 (2019). 31474532 10.1016/j.cub.2019.07.041

[5] P. Murúa, A. Garvetto, S. Egan, C. M. M. Gachon, The Reemergence of phycopathology: When algal biology meets ecology and biosecurity. Annu. Rev. Phytopathol. **61**, 231–255 (2023). 37253694 10.1146/annurev-phyto-020620-120425

[6] R. L. Naylor , A 20-year retrospective review of global aquaculture. Nature **591**, 551–563 (2021). 33762770 10.1038/s41586-021-03308-6

[7] P. D. Dawkins , Seagrass ecosystems as green urban infrastructure to mediate human pathogens in seafood. Nat. Sustain. **7**, 1247–1250 (2024).

[8] M. L. Vozzo , To restore coastal marine areas, we need to work across multiple habitats simultaneously. Proc. Natl. Acad. Sci. U.S.A. **120**, e2300546120 (2023). 37347794 10.1073/pnas.2300546120 PMC10293824

[9] B. L. Keeler , Linking water quality and well-being for improved assessment and valuation of ecosystem services. Proc. Natl. Acad. Sci. U.S.A. **109**, 18619–18624 (2012). 23091018 10.1073/pnas.1215991109 PMC3494932

[10] J. B. Lamb , Seagrass ecosystems reduce exposure to bacterial pathogens of humans, fishes, and invertebrates. Science **355**, 731–733 (2017). 28209895 10.1126/science.aal1956

[11] F. A. Ascioti, M. C. Mangano, C. Marcianò, G. Sarà, The sanitation service of seagrasses: Dependencies and implications for the estimation of avoided costs. Ecosyst. Serv. **54**, 101418 (2022).

[12] R. Unsworth, L. Cullen-Unsworth, B. Jones, R. Lilley, The planetary role of seagrass conservation. Science **377**, 609-617 2022). 35926055 10.1126/science.abq6923

[13] G. M. Ward , Ice-Ice disease: An environmentally and microbiologically driven syndrome in tropical seaweed aquaculture. Rev. Aquac. **14**, 414–439 (2022).

[14] M. Waycott , Accelerating loss of seagrasses across the globe threatens coastal ecosystems. Proc. Natl. Acad. Sci. U.S.A. **106**, 12377–12381 (2009). 19587236 10.1073/pnas.0905620106 PMC2707273

[15] R. R. Gentry , Exploring the potential for marine aquaculture to contribute to ecosystem services. Rev. Aquac. **12**, 499–512 (2020).

[16] E. A. Fiorenza , Data from “Seagrass ecosystems reduce disease risk and economic loss in marine farming production.” Dryad Digital. 10.5061/dryad.905qfttsz. Deposited 11 November 2024. PMC11670088 39680762

