# Supplementary material for: Seagrass ecosystems reduce disease risk and economic loss in marine farming production
Source: Proc Natl Acad Sci U S A. 2024 Dec 16;121(52):e2416012121. doi: 10.1073/pnas.2416012121 (PMC11670088; doi:10.1073/pnas.2416012121)
Supplement: Supplementary file 1 — Appendix 01 (PDF) [file pnas.2416012121.sapp.pdf]

Supporting Information for

Seagrass ecosystems reduce disease risk and economic loss in marine farming production

Evan A. Fiorenza, Nur Abu, William E. Feeney, Steven R. Limbong, Claire B. Freimark, Jamaluddin Jompa, C. Drew Harvell and Joleah B. Lamb

Corresponding Author: Joleah B. Lamb

Email: joleah.lamb@uci.edu

This PDF file includes:

Supporting Materials and Methods
Supporting References

Other supporting materials for this manuscript include the following:

Supporting Datasets S1–S6

Supporting Materials and Methods

Data availability

Datasets and code are available under https://doi.10.5061/dryad.905qfttsz.

Meta-analysis of bacterial diseases affecting seaweeds

We searched Web of Science for articles published between (1950 – 2020) using a search string to return all marine algal diseases:

TS = ((alga* OR seaweed OR microalga* OR macroalga* OR sea-weed* OR (sea AND weed*) OR kelp* OR cholorophyt* OR rhodophyt* OR ochrophyt* OR dinoflagellat* OR heterokont* OR cyanobacteria* OR prasinophyt* OR (micro AND alga*) OR (macro AND alga*) OR micro-alga* OR macro-alga*) AND(marine OR saltwater OR salt-water OR (salt AND water)) AND (disease* or parasit* or pathogen* or infect* or bleaching* or prevalen* or virus* or bacteri* or viral or fung* or nematod* or cestod* or trematod* or acanthoceph* or ectoparasit* or endoparasit* or worm* or protozoa* or protist* or myco* or chytrid* or epizoot* or vibrio* or (mass and mortalit*)) NOT (freshwater or (fresh and water) or fresh-water)).

A total of 11,349 articles were returned and screened for relevance in a stepwise process. We first screened titles and excluded articles that were clearly focused on pharmaceuticals, non-algal species, or in freshwater ecosystems. This resulted in 2,380 relevant articles whose abstracts were screened using the same criteria as the titles. A total of 1,099 articles remained whose full text were screened and from which data were extracted. From each relevant article, we extracted information on host and pathogen identity and geographic locations of empirical studies. Overall, we extracted data from 215 articles published between 1979 and 2020 that contained information on diseases of macroalgae. From the 215 articles, we filtered the reported host-pathogen pairs for those associated with bacteria in genera reported to be reduced in seagrass ecosystems from Lamb et al. (1) (*Dataset S1*). We further isolated all geographically reported incidences of disease in the seaweed *Kappaphycus* (*Dataset S2*).

Seagrass filtration mechanisms & background

Seagrass has been shown to regulate pathogens in several studies, suggesting that they offer a promising nature-based solution for mitigating disease outbreaks. For example, the presence of tropical seagrass meadows have been shown to be associated with 50% fewer waterborne bacteria that are potentially pathogenic to fishes, humans and invertebrates compared to paired seagrass absent sites (1). Subsequent studies have shown that this filtration service occurs regardless of the predominant seagrass species and whether they are tropical or temperate (2–4). Experimental studies have shown that bacterial pathogens are inhibited by phytochemicals extracted from seagrass tissues (5, 6), while cyanobacteria-dominated

biofilms that are found on seagrass blade surfaces and antimicrobial compounds from fungi that are associated with several seagrass species can specifically inhibit both fish and human pathogens (7). More recently, seagrass filtration services were conservatively estimated to prevent up to eight million cases of gastroenteritis each year globally (8).

Study locations and seaweed farming practices

Seagrass ecosystems are often removed for seaweed farming (9), providing a natural experiment to assess whether seagrass ecosystems influence disease levels in a co-cultivated marine resource. Fieldwork was conducted in Southwest Sulawesi, Indonesia, where seaweed farming is extensive (10). This region has a monsoonal climate, with an annual wet season from December to March and dry season from June to September. We conducted the study from 20 – 28 October 2015. A total of 16 farms were surveyed (*Dataset S3*), where seaweed is cultivated in coastal areas within seagrass ecosystems (n = 8 farms) and where seagrass ecosystems had been removed (n = 8 farms).

Seaweed surveys

Surveys were conducted at each of the 16 farms along three, randomly-selected 50 m line transects, with disease prevalence calculated as a binary observation for each individual seaweed. The number of seaweeds along the transect was recorded and identified to species level. Seaweeds were further categorized as: *diseased*, *compromised* (bleached or overgrown by ascidians, sponges, or algae), or *healthy* (no visual signs of disease of compromised health).

Seagrass cover and composition at the eight sites where seagrass ecosystems were not removed was estimated using three replicate 1 m x 1 m quadrats at three locations separated by approximately 20 m at each site. Seagrasses were identified to species level and percent cover was recorded (*Dataset S4*). Seagrass assemblages were similar among the eight sites where farmers cultivated alongside seagrass ecosystems and cover ranged from 39% to 92% (mean ± se = 72.3 ± 3.6). Five species of seagrass were identified across the eight sites. The most abundant species was *Enhalus acoroides*, which was present at all eight sites, with percent coverage ranging from 40 – 71%. *Thalassia hemprichii* was the second most abundant seagrass, present at five of the eight sites, and ranging in coverage from 6 – 14%. *Syringodium isoetifolium* and *Halophila ovalis* were each present at three sites, accounting for 2 – 6% of coverage at those sites. *Cymodocea rutundata* was the least abundant seagrass, present at only one site and accounting for 1% of coverage. Seaweed density did not differ significantly between sites where seagrass was present (12 individuals m^{-1}; range = 5-18 individuals m^{-1}) versus sites where seagrass was removed (10 individuals m^{-1}; range = 6-20 individuals m^{-1}, $t = 1.537$, $p = 0.131$).

Environmental parameters

In addition to opportunistic pathogens, environmental stressors are often associated with seaweed disease. Therefore, we assessed the association of eight environmental parameters with disease at each farm, including temperature (ºC), dissolved oxygen (mg L^{-1}), pH, salinity (ppt), total dissolved solids (mg L^{-1}), chlorophyll-α (μg L^{-1}), blue-green algae (phycocyanin cyanobacteria, RFU), and turbidity (formazin nephelometric units, FNU). Measurements were obtained by taking three replicate sample measurements at the center of three randomly selected lines of seaweed, at approximately 10 cm below the water surface using a multiparameter sonde (EXO2, Xylem Inc., New York, USA) (*Dataset S5*). We found a significant increase in five water quality parameters at farms where seagrass was present including temperature, salinity, dissolved oxygen, total dissolved solids, and pH compared to farms where seagrass was removed. We found a significant decrease in two water quality parameters at farms where seagrass was present including chlorophyll-α and blue-green algae. However, we found no associations between seaweed disease and these environmental parameters at farms where seagrass ecosystems were present compared to farms where seagrass was removed. We found no significant difference in levels of turbidity between seagrass present and removed treatments. However, among farms where seagrass was removed, seaweed disease prevalence was positively associated with turbidity (FNU range = 0.8 – 13.8), but this pattern was not observed at farms where seagrass was present (FNU range = 3.4 – 14.3, estimate ± standard error = 0.1 ± 0.009 versus 0.002 ± 0.01).

Productivity in seagrass meadows often peaks around the time of highest levels of photosynthetically available radiation (PAR) (11). We measured PAR using a quantum meter (Logan MQ-200, Apogee Instruments) and collected samples during approximately the same time of day during (11am – 2pm, PAR range = 1500 – 2100 μmol m^{-2} s^{-1}), which coincided with the lowest tidal fluctuations and water motion. Water flow velocity was measured using a flow probe (FP311, Global Water). Flow velocity was reduced by 45% within meadows (mean speed inside seagrass meadows = 4.14 m min^{-1} versus 7.35 m min^{-1} in paired control without seagrass, n = 6 each), which is consistent with other studies (12).

Enterococci are currently the only enteric indicator bacteria recommended by the U.S. Environmental Protection Agency for brackish and marine waters, since they correlate better with human health outcomes than other enteric indicator bacteria, such as fecal coliforms or *Escherichia coli* (13). Enterococci may also attach to aquatic vegetation and detritus (13), therefore we sampled carefully from surface waters in a small boat. Replicate samples were collected in 100 mL sterile bottles and immediately transported on ice to Hasanuddin University to be processed using a standard method for determining intestinal enterococci (ISO 7899-2 2000, (14)). Each sample was filtered using a sterile aseptic filter system (Millepore Sterifil, MA, USA) onto a 0.45 μm hydrophilic mixed cellulose ester GN-6 Metricel® gridded and sterile 47 mm membrane filter (Pall Life Sciences, MI, USA; lot no.

T32678). Filters were placed on sterile Slanetz and Bartley selective medium (Oxoid, Basingstoke, UK) inside 50 mm sterile petri dishes (Pall Life Sciences) and covered. Plates were immediately incubated at 37.0 ± 1.0 °C for 4.0 ± 0.5 hours followed by 44.0 ± 0.5 °C for 40 ± 4 hours and then counted. Fecal indicator bacteria (*Enterococcus*) was not found to differ between sites where seagrass was present (mean = 2.2 100 mL^{-1}; range = 0.3 – 8.6 100 mL^{-1}) versus sites where seagrass was removed (mean = 2.3 100 mL^{-1}, range = 0.6 – 3.3 100 mL^{-1}; $z = -0.287$, $p = 0.893$).

Statistical analyses

Datasets and code are available under https://doi.10.5061/dryad.905qfttsz.

All models were constructed and fit in the R statistical environment v4.4.0. Differences in seaweed disease prevalence was analyzed using a generalized linear mixed-effects model with a binomial error distribution and logit link (function *glmer()* in package *lme4*) (15). Seagrass status (present vs. removed) was treated as a fixed effect, while transect was nested into site (farm) and treated as a random effect to maintain consistency with the study design. The amount of diseased seaweed was offset according to the amount of healthy seaweed to account for the variation in sample size among replicates. Model selection was based on Akaike information criterion (AIC).

Differences in seaweed disease prevalence in relation to environmental parameters were analyzed using a generalized linear mixed-effects model with a binomial error distribution and logistic link (function *glm()* in package *lme4*) (15). To prevent overfitting of the model and determine which environmental parameter was most strongly associated with disease, we only included a single environmental parameter in each model. We also generated a correlation matrix to find closely related environmental parameters and generate a draftsman plot. Seagrass status (present vs. removed) and the environmental parameters were treated as fixed factors and allowed to interact. Site was not included as a random effect for this model set since the environmental parameters were included to explain the site-specific effects from the previous model set. The amount of diseased seaweed was offset according to the number of healthy seaweed to account for the variation in sample size among replicates. Model selection was based on AIC. Differences in environmental variables were analyzed using linear models (function *lm()* in package *stats*) (16). Seagrass status was treated as a fixed effect. We compared each model to the null model using a likelihood ratio test.

Potential for global co-cultivation of seagrass and seaweeds

To assess the potential for the co-cultivation of seagrasses and seaweeds, we investigated where seaweed aquaculture was possible and where seagrass ecosystems naturally occur. We followed methods for determining suitable areas for seaweed aquaculture from Froehlich et al. (17). Briefly, we obtained data

on surface nitrogen and phosphorus concentrations from the National Oceanic and Atmospheric Administration's (NOAA) World Ocean Atlas (18). For both nitrogen and phosphorus, we calculated the surface concentrations by averaging the concentrations of nitrogen and phosphorus at depths of 0m, 5m, and 10m. Using the average surface concentration, we created a ratio of N:P to determine suitable nutrient conditions. While Froelich et al. (17) used a minimum N:P ratio of 4:1 to determine the lower limit for nutrient conditions, this cutoff failed to capture areas of known seaweed aquaculture in Southeast Asia, therefore we didn't limit the lower end of nutrients. We then determined the average sea surface temperature for the period of 2008-2017 using NOAA CoRTAD v6 SST data (19). We restricted the temperature range to [20°C, 35°C] for *Kappaphycus* production as this range is the thermal tolerance limits for *Kappaphycus* (20, 21). We used this temperature layer to limit the suitable N:P ratios to those that also fell within suitable temperature ranges (function *mask(),* package *raster*) (22). We then only included areas that fell within exclusive economic zones as we assumed that cultivation will not occur in the high seas. We then incorporated seagrass species distributions obtained from the International Union for the Conservation of Nature (IUCN) (23) and masked the suitable area for aquaculture with known seagrass distributions to obtain the area where seaweed farming can occur near seagrass ecosystems.

Potential economic value of disease risk reduction

To estimate the value (V) of co-cultivation (C) of *Kappaphycus* (K) and seagrasses (S), we assumed that there are two scenarios, one where seagrasses were removed (NS) in favor of *Kappaphycus* production and one where co-cultivation was practiced. The difference between the estimated values of the produced *Kappaphycus* for both scenarios would then provide the added value of co-cultivation (Equation 1).

$$V(C) \sim V(K|S) - V(K|NS) \tag{1}$$

To estimate the value of the produced *Kappaphycus* in the two scenarios, we first needed to determine potential *Kappaphycus* production per area (*P*). We simulated the potential density (d) of individual plants per meter of culture line using a normal distribution with a mean density and standard error calculated from the density of algal individuals from disease surveys to create a vector of total (both healthy and diseased) algal density for either scenario. We then generated potential disease prevalence under two scenarios, one with seagrass co-cultivation and one without using data collected from disease surveys. For this, we calculated the mean prevalence and error from disease surveys and then subtracted this from one to calculate the prevalence of healthy seaweed. We then multiplied the simulated healthy seaweed prevalence (p) by the simulated seaweed density to generate a per meter density of viable seaweed with and without co-cultivation, assuming that all diseased individuals are lost through mechanical breakage or

reduction in value and thus do not contribute to profit. We then converted the density of plants to wet weight, assuming that plants were harvested at 1 kg (weight at harvest - w, (24)). We then converted the wet weight production per meter of line to a dry weight at a conversion factor of 0.1 (wet to dry weight conversion - ξ, (25)). Then, using the assumption that *Kappaphycus* lines are normally 0.7m apart (line spacing - ς,(26)), we calculated the dry weight production per meter squared (Equation 2).

$$P_X = d(K|X) * (1 - p(x)) * w * \xi * \frac{1}{\varsigma}$$
$$X: \{S, NS\}$$

[2]

Then, we estimated the farm-gate value of dry *Kappaphycus* using the average of six farm gate prices available from different countries in 2015, excluding inflation and the potential for other uses of *Kappaphycus* that would change prices ($\overline{G}$, (27)). We then multiplied the average farm-gate value by the production of dry *Kappaphycus* per meter squared to calculate a potential revenue per meter squared per harvest. We calculated this for both scenarios and calculated the difference to estimate the added value of seagrass in co-cultivation at the per square kilometer scale and if all potential co-culturable areas were utilized (Equation 3).

$$V(X) = P_X * \overline{G}$$
$$X: \{S, NS\}$$

[3]

As growing season length varies based on location, annual revenue can be calculated by multiplying the revenue per harvest by the number of harvests per year. For our calculations, we assumed only a single harvest per year to be conservative in our estimates (*Dataset S6*).

Supporting Datasets

Supplementary data are available under https://doi.10.5061/dryad.905qfttsz.

Dataset S1. (separate file)

Causative agents of diseases that affect macroalgae by bacterial pathogens shown to be reduced by seagrass ecosystems.

Dataset S2. (separate file)

Reported global locations of diseases affecting seaweed in the genus *Kappaphycus*.

Dataset S3. (separate file)

Farm study locations.

Dataset S4. (separate file)

Seagrass cover and composition by species at farm study locations.

Dataset S5. (separate file)

Environmental variables at seaweed farming locations where seagrass is present or seagrass removed. Each variable was recorded in triplicate per location.

Dataset S6. (separate file)

Modeled co-cultivation area of seagrass ecosystems and suitable seaweed conditions per country or associated territory and mean potential revenue.

Supporting References

1. J. B. Lamb, *et al.*, Seagrass ecosystems reduce exposure to bacterial pathogens of humans, fishes, and invertebrates. *Science* **355**, 731–733 (2017).
2. T. B. H. Reusch, *et al.*, Lower Vibrio spp. abundances in *Zostera marina* leaf canopies suggest a novel ecosystem function for temperate seagrass beds. *Mar. Biol.* **168**, 149 (2021).
3. Y. Deng, S. Liu, J. Feng, Y. Wu, C. Mao, What drives putative bacterial pathogens removal within seagrass meadows? *Mar. Pollut. Bull.* **166**, 112229 (2021).
4. P. D. Dawkins, *et al.*, Seagrass ecosystems as green urban infrastructure to mediate human pathogens in seafood. *Nat. Sustain.* 1–4 (2024).
5. R. R. R. Kannan, R. Arumugam, P. Anantharaman, Antibacterial potential of three seagrasses against human pathogens. *Asian Pac. J. Trop. Med.* **3**, 890–893 (2010).
6. C. S. Kumar, D. V. Sarada, T. P. Gideon, R. Rengasamy, Antibacterial activity of three South Indian seagrasses, *Cymodocea serrulata, Halophila ovalis and Zostera capensis*. *World J. Microbiol. Biotechnol.* **24**, 1989–1992 (2008).
7. P. Supaphon, S. Phongpaichit, V. Rukachaisirikul, J. Sakayaroj, Antimicrobial potential of endophytic fungi derived from three seagrass species: C*ymodocea serrulata, Halophila ovalis and Thalassia hemprichii*. *PloS One* **8**, e72520 (2013).
8. F. A. Ascioti, M. C. Mangano, C. Marcianò, G. Sarà, The sanitation service of seagrasses – Dependencies and implications for the estimation of avoided costs. *Ecosyst. Serv.* **54**, 101418 (2022).
9. M. de la Torre-Castro, P. Rönnbäck, Links between humans and seagrasses—an example from tropical East Africa. *Ocean Coast. Manag.* **47**, 361–387 (2004).
10. A. Q. Hurtado, I. C. Neish, A. T. Critchley, Developments in production technology of *Kappaphycus* in the Philippines: more than four decades of farming. *J. Appl. Phycol.* **27**, 1945–1961 (2015).
11. P. Berg, *et al.*, Dynamics of benthic metabolism, O2, and pCO2 in a temperate seagrass meadow. *Limnol. Oceanogr.* **64**, 2586–2604 (2019).
12. M. E. Anderson, J. M. Smith, S. McKay, Wave Dissipation by Vegetation (2011).
13. M. N. Byappanahalli, M. B. Nevers, A. Korajkic, Z. R. Staley, V. J. Harwood, Enterococci in the Environment. *Microbiol. Mol. Biol. Rev.* **76**, 685–706 (2012).
14. ISO 7899-2, Water quality - Detection and enumeration of intestinal enterococci - Part 2: membrane filtration method. *International Organization for Standardization, Geneva, Switzerland* (2000).
15. D. Bates, M. Maechler, B. Bolker, S. Walker, Fitting linear mixed-effects model using lme4. *Journal of Statistical Software* **67**, 1–48 (2015).
16. R Core Team, R: A language and environment for statistical computing. *R Foundation for Statistical Computing, Vienna, Austria* (2021).
17. H. E. Froehlich, J. C. Afflerbach, M. Frazier, B. S. Halpern, Blue growth potential to mitigate climate change through seaweed offsetting. *Curr. Biol.* **29**, 3087-3093.e3 (2019).
18. H. E. Garcia, *et al.*, World Ocean Atlas 2013. Volume 4, Dissolved Inorganic Nutrients: Phosphate, Nitrate, Silicate. NOAA Atlas NESDIS 76. U.S. Department of Commerce, National Oceanic and Atmospheric Administration (2013).
19. K. Saha, *et al.*, The coral reef temperature anomaly database (CoRTAD) Version 6 - Global, 4km Sea Surface Temperature and Related Thermal Stress Metrics for 1982 to 2019. NOAA National Centers for Environmental Information. Dataset. https://doi.org/10.25921/ffw7-cs39.
20. I. A. Borlongan, G. S. Gerung, S. Kawaguchi, G. N. Nishihara, R. Terada, Thermal and PAR effects on the photosynthesis of *Eucheuma denticulatum* and *Kappaphycus striatus* (so-called Sacol strain) cultivated in shallow bottom of Bali, Indonesia. *J. Appl. Phycol.* **29**, 395–404 (2017).
21. Lideman, G. N. Nishihara, T. Noro, R. Terada, Effect of temperature and light on the photosynthesis as measured by chlorophyll fluorescence of cultured *Eucheuma denticulatum* and *Kappaphycus sp.* (Sumba strain) from Indonesia. *J. Appl. Phycol.* **25**, 399–406 (2013).
22. R. Hijmans, raster: Geographic Data Analysis and Modeling. R package version 3.5-29 (2022).
23. IUCN, The IUCN Red List of Threatened Species. version 2019. (2019).
24. J. Collen, M. Mtolera, K. Abrahamsson, A. Semesi, M. Pedersen, Farming and Physiology of the red algae *Eucheuma*: Growing commercial importance in East Aftrica. *Ambio* **24**, 497–501 (1995).
25. C. M. Duarte, J. Wu, X. Xiao, A. Bruhn, D. Krause-Jensen, Can seaweed farming play a role in climate change mitigation and adaptation? *Front. Mar. Sci.* **4** (2017).
26. Q. Yang, *et al.*, Potential use of mangroves as constructed wetland for municipal sewage treatment in Futian, Shenzhen, China. *Mar. Pollut. Bull.* **57**, 735–743 (2008).
27. D. Valderrama, *et al.*, The Economics of *Kappaphycus* seaweed cultivation in developing countries: A Comparative analysis of farming systems. *Aquac. Econ. Manag.* **19**, 251–277 (2015).
